# Recombinant irisin prevents cell death and mineralization defects induced by random positioning machine exposure in primary cultures of human osteoblasts: A promising strategy for the osteoporosis treatment

**DOI:** 10.3389/fphys.2023.1107933

**Published:** 2023-03-15

**Authors:** Ida Cariati, Roberto Bonanni, Anna Maria Rinaldi, Mario Marini, Riccardo Iundusi, Elena Gasbarra, Virginia Tancredi, Umberto Tarantino

**Affiliations:** ^1^ Department of Clinical Sciences and Translational Medicine, “Tor Vergata” University of Rome, Rome, Italy; ^2^ Department of Systems Medicine, “Tor Vergata” University of Rome, Rome, Italy; ^3^ Department of Orthopaedics and Traumatology, “Policlinico Tor Vergata” Foundation, Rome, Italy; ^4^ Centre of Space Bio-Medicine, “Tor Vergata” University of Rome, Rome, Italy

**Keywords:** random positioning machine, bone loss, apoptosis, cell viability, mineralization, simulated microgravity, recombinant irisin

## Abstract

Spaceflight exposure, like prolonged skeletal unloading, is known to result in significant bone loss, but the molecular mechanisms responsible are still partly unknown. This impairment, characterizing both conditions, suggests the possibility of identifying common signalling pathways and developing innovative treatment strategies to counteract the bone loss typical of astronauts and osteoporotic patients. In this context, primary cell cultures of human osteoblasts derived from healthy subjects and osteoporotic patients were exposed to random positioning machine (RPM) to reproduce the absence of gravity and to exacerbate the pathological condition, respectively. The duration of exposure to RPM was 3 or 6 days, with the aim of determining whether a single administration of recombinant irisin (r-irisin) could prevent cell death and mineralizing capacity loss. In detail, cellular responses were assessed both in terms of death/survival, by MTS assay, analysis of oxidative stress and caspase activity, as well as the expression of survival and cell death proteins, and in terms of mineralizing capacity, by investigating the pentraxin 3 (PTX3) expression. Our results suggest that the effects of a single dose of r-irisin are maintained for a limited time, as demonstrated by complete protection after 3 days of RPM exposure and only partial protection when RPM exposure was for a longer time. Therefore, the use of r-irisin could be a valid strategy to counteract the bone mass loss induced by weightlessness and osteoporosis. Further studies are needed to determine an optimal treatment strategy based on the use of r-irisin that is fully protective even over very long periods of exposure and/or to identify further approaches to be used in a complementary manner.

## 1 Introduction

Space exploration represents one of the greatest adventures mankind has faced since the second half of the 20th century. However, the harsh environment of outer space, characterized by microgravity and radiation, poses significant health risks for astronauts, with a damaging impact especially on the musculoskeletal system (Tominari et al., 2019; Tarantino et al., 2020). Indeed, long-term space missions have been reported to cause bone demineralization and muscle atrophy, promoting the onset of osteoporosis and sarcopenia, as well as cardiovascular deconditioning and immune dysfunction (Prasad et al., 2020; Cariati et al., 2022b). Despite the knowledge advancement in this field, many of these pathophysiological changes still cannot be adequately counteracted by exercise or nutritional supplementation alone, suggesting the need for further studies to better elucidate the underlying mechanisms (Palmieri et al., 2019; Sandal et al., 2020; Cariati et al., 2021a; Hughes et al., 2022).

In this context, the reactive oxygen species (ROS) generation and the resulting oxidative stress have been proposed to be among the main contributors to the bone mass loss occurring in real and simulated microgravity conditions, suggesting the use of appropriate antioxidant countermeasures as a viable strategy to prevent weightlessness-induced alteration of bone homeostasis (Tian et al., 2017). Specifically, Morikawa et al. found that mechanical unloading significantly increased intracellular ROS production in a mouse model of tail suspension, exacerbating bone loss due to reduced osteoblastic capacity. However, vitamin C administration significantly attenuated bone loss during unloading due to its antioxidant action (Morikawa et al., 2013). Similarly, Xin and others suggested curcumin treatment as an effective countermeasure to inhibit discharge-induced ROS formation, enhancing osteoblastic differentiation and attenuating osteoclastogenesis in rats exposed to limb unloading (Xin et al., 2015). More recently, Morabito et al. demonstrated that treatment with 6-hydroxy-2,5,7,8-tetramethylchroman-2-carboxylic acid (Trolox), a water-soluble vitamin E analogue, counteracted oxidative damage induced by prolonged exposure to random positioning machine (RPM) in a murine osteoblast cell line, also preserving the cell cytoskeleton architecture and restoring cell proliferation rate and metabolism (Morabito et al., 2020).

Altered calcium metabolism is also among the mechanisms proposed to explain the demineralizing effects observed in astronauts after short- and long-term space missions (Sun et al., 2019). Particularly, excessive calcium release from bone tissue is known to occur in weightlessness, causing suppression of parathyroid hormone (PTH) and reduction of circulating 1,25-dihydroxyvitamin D (1,25(OH)2D), both of which are responsible for reduced calcium absorption. Thus, the bone mass loss and muscle wasting are associated with a drastic reduction in intestinal calcium absorption, which promotes bone resorption and the subsequent onset of osteoporosis (Grimm et al., 2016). In this regard, the effects of prolonged exposure to RPM on the mineralizing capacity and expression of pentraxin 3 (PTX3), a positive regulator of mineralization and bone formation, in the human osteosarcoma cell line SAOS-2 were recently investigated. Interestingly, RPM exposure was observed to have a strong impact on the mineralization process, as evidenced by the reduced presence of calcifying nodules, calcium deposits and PTX3 expression (Cariati et al., 2022a). In agreement, Hu and others found a significant reduction in osteoblastic differentiation and mineralized nodule formation in murine MC3T3-E1 preosteoblasts exposed to RPM for 24 h, in association with reduced expression of certain differentiation markers, such as runt-related transcription factor 2 (RUNX2), osteocalcin (OCN) and type I collagen (Hu et al., 2015). Finally, the inhibitory effects of microgravity on osteoblast function have recently been confirmed by Braveboy-Wagner and Lelkes, who observed that exposure of 7F2 osteoblasts to various levels of simulated partial gravity resulted in a significant gravity-dependent inhibition of short-term (6 days) proliferation and alkaline phosphatase (ALP) activity and long-term (21 days) mineralization, suggesting a close association between impaired cell function and simulated partial gravity levels (Braveboy-Wagner and Lelkes, 2022). However, despite numerous research efforts in recent years, the mechanisms by which weightlessness causes high bone resorption and a rapid decrease in bone minerals and calcium are largely unknown, as is the knowledge about the role of potential markers in microgravity-dependent bone tissue loss. Undoubtedly, studying the mechanisms responsible for cellular adaptations to no load and simulated microgravity could improve our knowledge of the bone loss typical of musculoskeletal diseases induced by disuse or prolonged bedding, such as osteoporosis, paving the way for new therapeutic strategies to counteract osteogenic and mineralizing deficits. In this respect, an in-depth investigation of the molecular mechanisms involved in the pathogenesis of the disease and how these are altered by prolonged periods of sedentary lifestyle appears to be necessary to develop optimal treatment strategies to counteract the bone loss found both in astronauts and in subjects, osteoporotic or not, who are forced into a sedentary lifestyle.

Interestingly, the strong impact of RPM exposure on mineralization has been proposed to depend on an alteration of osteoblast proliferation and apoptosis processes. In this regard, Bucaro et al. (2004) exposed MC3T3-E1 cells to clinostat treatment to assess changes in the expression of B-cell lymphoma 2 (Bcl-2) and Akt/protein kinase B (PKB), two important regulators of apoptosis and cell survival. In association with a reduction in the key bone markers expression, clinostat exposure was observed to cause a marked downregulation of Bcl-2 and Akt, highlighting the strong impact of weightlessness on cell proliferation and apoptosis. Subsequently, Dai and others also confirmed the Akt deregulation in rat bone marrow mesenchymal stem cells (rBMSCs), showing a reduction in Akt phosphorylation levels after 3 days of clinorotation (Dai et al., 2007). Therefore, an altered expression of key regulators of cell death, survival and proliferation could be responsible for the osteogenesis and mineralization defects induced by the absence of load.

Noteworthy, irisin has recently been proposed as an important regulator of bone metabolism. This interesting 112 amino acid hormone represents the cleavage product of the transmembrane precursor fibronectin type III domain-containing protein 5 (FNDC5) and, once secreted, modulates numerous physiological adaptations to exercise (Liu et al., 2021). In this regard, Colaianni et al. (2017) suggested the use of recombinant irisin (r-irisin) as a tool to restore bone loss and muscle atrophy in mice subjected to limb unloading. Specifically, microCT analysis of femurs showed that r-irisin administration preserved both cortical and trabecular bone mineral density (BMD), as well as protecting against muscle mass decline, suggesting the irisin-based therapy development as a viable strategy for both elderly patients with osteoporosis and immobile physical disabilities and astronauts subjected to microgravity-induced bone and muscle loss. In agreement, Colucci and others assessed the mRNA levels of genes coding for important markers of bone metabolism, such as RUNX2, osteoprotegerin, collagen I and osterix, in a 3D cell model exposed to 14 days of microgravity aboard the SpaceX Dragon cargo ferry to the international space station, observing significant downregulation. Interestingly, r-irisin treatment prevented this effect, in association with an increase in osteoprotegerin expression, suggesting r-irisin as a supportive tool for osteoblastic differentiation and activity in microgravity, as well as a countermeasure to prevent weightlessness-induced bone loss (Colucci et al., 2020). The mechanisms by which irisin acts as a positive regulator in bone formation under microgravity conditions are largely unknown. However, Chen and others recently proposed that r-irisin could positively regulate osteoblast differentiation under simulated microgravity conditions by increasing the β-catenin expression and promoting the expression of osteogenic markers, such as ALP and collagenIα1 (colIα1), and cell proliferation-related genes, such as cyclin-dependent kinases (CDKs) 2 and 12 and cyclins A2, D1 and E1 (Chen et al., 2020). Although the effects of irisin on multiple organs and tissues are well known and widely documented, this regulator of bone metabolism has recently been the subject of debate. Particularly, Albrecht et al. highlighted a contradiction in the data regarding the existence of circulating irisin and its role in human and other animal metabolism, calling it a myth rather than an exercise-induced myokine (Albrecht et al., 2015). This controversy over the existence and efficacy of irisin lays the groundwork for more in-depth investigations aimed at assessing its protective action against damage induced by weightlessness.

Considering that prolonged skeletal unloading through bed rest is known to induce bone loss like that seen in elderly osteoporotic patients, but with an accelerated time lapse, and that this same effect is also observed in astronauts exposed to spaceflight (Camirand et al., 2016), the aim of our work was to test the efficacy of a single administration of r-irisin in counteracting the cell death and bone demineralization common to these conditions. To this end, we (i) exposed primary cultures of human osteoblasts obtained from healthy subjects and osteoporotic patients to RPM to mimic weightlessness and to exacerbate the pathological condition, respectively, and (ii) determined, through exposure to RPM for 3 or 6 days, the potential effects of irisin as a therapeutic strategy to counteract the bone loss characterizing astronauts and osteoporotic patients.

## 2 Materials and methods

### 2.1 Participants

A total of 30 individuals admitted to the Orthopaedic Department of the Policlinico “Tor Vergata” were enrolled in this study and divided into two experimental groups: a healthy group consisting of 15 subjects undergoing hip arthroplasty for high-energy hip fracture (HEALTHY), and a group of 15 patients undergoing hip arthroplasty for fragility fracture (OP).

Subjects with endocrine disorders affecting mineral and bone metabolism, myopathies or other neuromuscular diseases, cancer, chronic viral infections, and diabetes, as well as those undergoing chronic administration of corticosteroids for autoimmune diseases or previous orthopaedic surgical implants were excluded from the study. All participants obtained informed consent prior to surgery.

### 2.2 Clinical parameters

The densitometric diagnosis of osteoporosis based on dual-energy X-ray absorptiometry (DXA) assessment of BMD was performed in each patient using a Lunar DXA apparatus (GE Healthcare, Madison, WI, United States). According to the manufacturer’s recommendations, scans of the lumbar spine (L1-L4) and femur (neck and total) were performed, and BMD was measured (in grams per square centimeter) with a coefficient of variation of 0.7% on the uninjured limb (Celi et al., 2013). For both experimental groups, DXA assessment was performed 1 month postoperatively, and results were expressed as *T*-scores.

### 2.3 Specimen collection

Femoral head biopsies were collected from each participant during hip arthroplasty surgery. The specimens were subsequently processed for setting up of primary osteoblast cultures and subjected to qualitative and quantitative evaluations. Sample processing was conducted according to approved guidelines. Moreover, all experimental procedures described in this study were approved by the Ethics Committee of the Policlinico “Tor Vergata” (approval reference number #17/21) and were performed according to the World Medical Association’s Code of Ethics (Declaration of Helsinki).

### 2.4 Isolation and culture of primary human osteoblastic cells

Primary cultures of osteoblasts were set up from trabecular bone fragments taken during hip arthroplasty surgery. Specifically, the fragments were first washed in phosphate buffered saline (PBS) and then incubated at 37°C with 1 mg/mL porcine pancreatic trypsin ≥60 U/mg (SERVA Electrophoresis GmbH Heidelberg, DE) diluted in PBS. Subsequently, bone fragments were subjected to repeated digestions with 2.5 mg/mL collagenase NB 4G Proved grade ≥0.18 U/mg (SERVA Electrophoresis GmbH, Heidelberg, DE) diluted in PBS with calcium and magnesium. After digestions completion, the supernatant was collected and centrifuged at 340 RCF for 10 min. Cells were seeded into a 24-well plate at a density of 2 × 10^4^ cells/well and maintained in DMEM-F12 (Biowest SAS, Nuaillé, France) supplemented with 10% fetal bovine serum (FBS) (Biowest SAS, Nuaillé, France) growth medium, 100 Units/mL penicillin and 100 μg/mL streptomycin (Sigma-Aldrich, St. Louis, MO, United States) and 2 mmol/L stable glutamine (Biowest SAS, Nuaillé, France) in an incubator at 37°C, 5% CO_2_ until reaching confluence. The medium was changed every 3–4 days. During RPM exposure, the growth medium was supplemented with 50 μg/mL ascorbic acid (Sigma-Aldrich, St. Louis, MO, United States) and 10 mM β-glycerophosphate (Sigma-Aldrich, St. Louis, MO, United States).

### 2.5 Simulation experiment by RPM

The biological effects of microgravity were simulated in primary cultures of human osteoblasts using the RPM system (Airbus Defence and Space Netherlands B.V.), according to the previously described procedures (Wuest et al., 2015; Cariati et al., 2022b). The rotating RPM frame was placed inside a standard CO_2_ cell culture incubator. The RPM movements were controlled by software with a personalized algorithm, which rotated with a random speed such that the mean gravity vector converged to zero over time. Centrifugal acceleration and artefacts were minimized by placing cell samples in the centre of the rotation using 24-well plates. A dialysis membrane (Visking Medicell International Ltd. Liverpool Road-London code DTV12000.06.000 MWCO 12/14 KDa) was deposited on the convex liquid meniscus of the culture medium within each well to seal it and prevent the air bubbles formation. The nitrocellulose discs were fixed to the support by means of a rubber ring to minimize the effects of flow shear on the attached cells.

Primary cultures of human osteoblasts were exposed to RPM for 3 days or 6 days. Other plates were exposed to a normogravity regime, keeping them in the incubator for the same period so that all cell samples shared the same experimental conditions.

### 2.6 Primary cultures of human osteoblasts conditioned with r-irisin

The role of irisin in preventing damage induced by RPM exposure was investigated by treating primary cultures of human osteoblasts with r-irisin. Specifically, cells from the first or second passage were seeded in a 24-well plate at a density of 2 × 10^4^ cells/well. Primary cultures derived from each patient group were incubated with 10 ng/mL r-irisin (AG-20B-0153, AdipoGen^®^ Life Sciences, Liestal, Switzerland) and exposed to RPM for 3 days or 6 days. Subsequently, the r-irisin-treated cell cultures were subjected to the same experimental procedures as the untreated samples.

### 2.7 Cell viability assessment

Cell viability was investigated using CellTiter 96 AQueous One (Promega, Madi-son, WI, United States), a colorimetric method for identifying viable cells (Kabakov and Gabai, 2018; Cariati et al., 2021b). The CellTiter 96 AQueous Assay consists of a novel tetrazolium compound (3-(4,5-dimethylthiazol-2-yl)-5-(3-carboxymethoxyphenyl)-2-(4-sulfophenyl)-2H-tetrazolium-MTS) and an electron-coupling reagent (phenazinemethosulfat-PMS). MTS is bioreduced by cells into a formazan product that is soluble in tissue culture medium. The absorbance of the formazan at 490 nm can be measured directly from 96-well assay plates without additional processing. Briefly, 20 µL of MTS/PMS solution was added to 100 µL of hank’s balanced salt solution (HBSS) in each well and incubated for at least 2 h at 37°C. The final concentrations of MTS and PMS were 333 μg/mL and 25 μM, respectively. The quantity of formazan product as measured by the amount of 490 nm (Spark Multimode Microplate Reader—Tecan, Austria) absorbance is directly proportional to the number of living cells in the culture. For each condition, the experiment was conducted in quadruplicate (*n* = 20 from *N* = 5 experiments).

### 2.8 Measurement of intracellular ROS level

Changes in intracellular ROS level induced by RPM exposure in primary osteoblast cultures were detected using the fluorescent probe 2',7'-dichlorodihydrofluorescein di-acetate (H2DCFDA) (D399, InvitrogenTM, ThermoFisher Scientific, United States). After RPM exposure, all cell samples were washed with PBS several times and incubated with 10 μM H2DCFDA for 40 min at 37°C in the dark. The mean fluorescence intensity of each experimental group was measured using a plate reader (Spark Multimode Microplate Reader—Tecan, Austria), which represented the intracellular ROS level (Xin et al., 2015). For each condition, the experiment was conducted in quadruplicate (*n* = 20 from *N* = 5 experiments).

### 2.9 Caspase activity assay for apoptosis detection

Caspase activity was determined by luminescence using the Caspase-Glo 3/7 Assay (Promega, Madison, WI, United States), as indicated by the manufacturer. The assay provides a luminogenic caspase-3/7 substrate containing the DEVD tetrapeptide sequence in a reagent optimized for caspase activity, luciferase activity, and cell lysis. Caspase-Glo 3/7 reagent causes cell lysis, followed by cleavage of the substrate by caspase and generation of a luminescent signal produced by luciferase. The luminescence is proportional to the amount of caspase activity present. This assay provides an accurate, rapid, and sensitive measurement of caspase 3/7 activity. Before use, the kit components, Caspase-Glo Buffer and lyophilized Caspase-Glo 3/7 Substrate, were equilibrated at room temperature. Then, the Caspase-Glo Buffer was transferred inside the bottle containing the lyophilized substrate and the contents were gently shaken until the substrate was completely dissolved. Caspase-Glo Reagent 3/7 thus constituted was added to each well in a 1:1 ratio, and the multiwell was shaken gently for about 30 s. After the 3-h incubation at constant temperature, the luminescence of each sample was determined using a plate reader (Spark Multimode Microplate Reader—Tecan, Austria). For each condition, the experiment was conducted in triplicate (*n* = 9 from *N* = 3 experiments).

### 2.10 Immunocytochemistry

An immunocytochemistry was performed to characterize the primary osteoblast cultures by analyzing the RUNX2 expression, as well as to study the mineralization process by analyzing the PTX3 expression in all experimental groups. After fixation in 4% paraformaldehyde for 15 min, the cell samples were pre-treated with EDTA citrate (pH 7.8) for 30 min at 95°C and then incubated for 1 h with rabbit monoclonal anti-RUNX2 (#12556 Cell Signalling Technology) or rat monoclonal anti-PTX3 (clone MNB1, AbCam). Washings were performed with PBS/Tween20 (pH 7.6) (UCS Diagnostic, Rome, Italy). The immunocytochemical reaction was detected using the horse-radish peroxidase (HRP)-3,3′diaminobenzidine (DAB) detection kit (UCS Diagnostic, Rome, Italy). Specifically, 50 μL of DAB/450 μL of substrate were incubated for 3 min. To assess the immunostaining background, we included negative controls for each reaction by incubating sections with secondary antibodies (HRP) alone or a detection system (DAB) alone (Supplementary Figures S1, S2). Immunopositive cells for RUNX2 and PTX3 were detected using NIS-Elements software (5.30.01; Laboratory Imaging, Prague, Czech Republic) and expressed as a percentage of the total analyzed for RUNX2 and PTX3. For each condition, the experiment was conducted in triplicate (*n* = 9 from *N* = 3 experiments).

### 2.11 Western blotting analysis

The expression of Akt, Bcl-2, Bax and PTX3 was detected in primary cultures of human osteoblasts by Western blotting analysis. Cell proteins extracted by using RIPA buffer were separated by 8%–16% precast SDS-PAGE (Bio-Rad, Hercules, CA, United States) under reduced conditions. Protein concentration was determined using the Pierce BCA Protein Assay Kit (Thermo Scientific, Vilnius, Lithuania). Equal amounts of protein (20 μg for Akt and PTX3, 25 μg for Bcl-2 and Bax) were resolved on 8%–16% precast SDS-PAGE and transferred to PVDF membrane. Then membranes were incubated with rabbit mono-clonal anti-Akt (#4685 Cell Signalling Technology), mouse monoclonal anti-Bcl-2 (#15071 Cell Signalling Technology), mouse monoclonal anti-Bax (sc-7480 Santa Cruz Biotechnology, Inc.), or rat monoclonal anti-PTX3 (clone MNB1, AbCam) and successively with anti-rabbit IgG coupled to HRP, anti-mouse IgG coupled to HRP, or anti-rat IgG coupled to HRP, respectively. In addition, the same membranes were incubated with mouse monoclonal anti-GAPDH (ab8245, AbCam), used for normalization. Immunoreactive electrophoretic bands were detected by enhanced chemiluminescence (ECL Advance, Amersham; GE Healthcare Life Sciences, Little Chalfont, Buckinghamshire, United Kingdom) using a VersaDoc 5,000 Imager (Bio-Rad). The expression of Akt and PTX3, as well as the Bcl-2/Bax ratio, under the different experimental conditions were quantified by calculating the densitometric values of the relevant bands and normalizing the results against the expression of GAPDH, expressing them as mean ± standard error. The original Western blotting images are shown in Supplementary Figure S3.

### 2.12 Statistical analysis

All statistical analyses were performed using GraphPad Prism 8 software (GraphPad Prism 8.0.1, La Jolla, CA, United States). For all experimental procedures, data were expressed as mean ± standard error. Furthermore, data were compared by one-way ANOVA and Tukey’s multiple comparison test and were considered significantly different if *p* < 0.05.

## 3 Results

### 3.1 Clinical evaluation

The participants enrolled in this study were divided into two experimental groups: 15 healthy subjects undergoing hip arthroplasty for high-energy hip fracture (HEALTHY) and 15 patients undergoing hip arthroplasty for fragility fracture (OP). All of them were characterized by clinical and instrumental evaluation. Table 1 shows the parameters analyzed for each group of participants, which include age (years), bone mass index (BMI), *T*-score (L1–L4), *T*-score (femoral neck), and *T*-score (total femur).

**TABLE 1:** Clinical characteristics of HEALTHY subjects and OP patients.

Parameters	HEALTHY (*n* = 15)	OP (*n* = 15)	Significance
Age (years)	47.4 ± 2.8	77.3 ± 2.7	***p < 0.001
BMI (Kg/cm^2^)	25.6 ± 2.4	22.1 ± 3.1	NS (*p* = 0.316)
*T*-score (L1–L4)	0.4 ± 0.6	−2.5 ± 0.3	***p < 0.001
*T*-score (femoral neck)	0.9 ± 0.1	−2.6 ± 0.3	***p < 0.001
*T*-score (total femur)	1.0 ± 0.9	−2.4 ± 0.2	***p < 0.001

HEALTHY: subjects underwent hip arthroplasty for high-energy hip fracture; OP: patients underwent hip arthroplasty for fragility fracture; BMI: bone mass index; ***: *p* < 0.001; NS: no significance.

HEALTHY subjects and OP patients differ in terms of median age (47.4 ± 2.8 vs. 77.3 ± 2.7, ***p < 0.001). BMD assessment of the lumbar spine, femoral neck, and total femur, expressed as *T*-score values, showed a statistically significant difference between the two groups (***p < 0.001). In fact, values of 0.4 ± 0.6, 0.9 ± 0.1 and 1.0 ± 0.9 were recorded for *T*-score (L1–L4), *T*-score (femoral neck), and *T*-score (total femur), respectively, in the HEALTHY group. In contrast, OP patients were characterized by *T*-score (L1–L4), *T*-score (femoral neck), and *T*-score (total femur) values of −2.5 ± 0.3, −2.6 ± 0.3, and −2.4 ± 0.2, respectively. Moreover, no significant differences were found for BMI values between the HEALTHY (25.6 ± 2.4) and OP (22.1 ± 3.1) groups.

### 3.2 RPM exposure influences survival of primary human osteoblastic cells

The effects of two different exposure times to RPM, specifically 3 or 6 days, on primary cultures of human osteoblasts from HEALTHY subjects and OP patients were assessed in terms of cell viability by MTS assay; oxidative stress by measurement of intracellular ROS level; apoptosis detection by caspase-3/7 activity assay; and expression of Akt, Bcl-2, and Bax, three important regulators of cell survival and death, by Western blotting analysis.

#### 3.2.1 HEALTHY subjects

**FIGURE 1:**
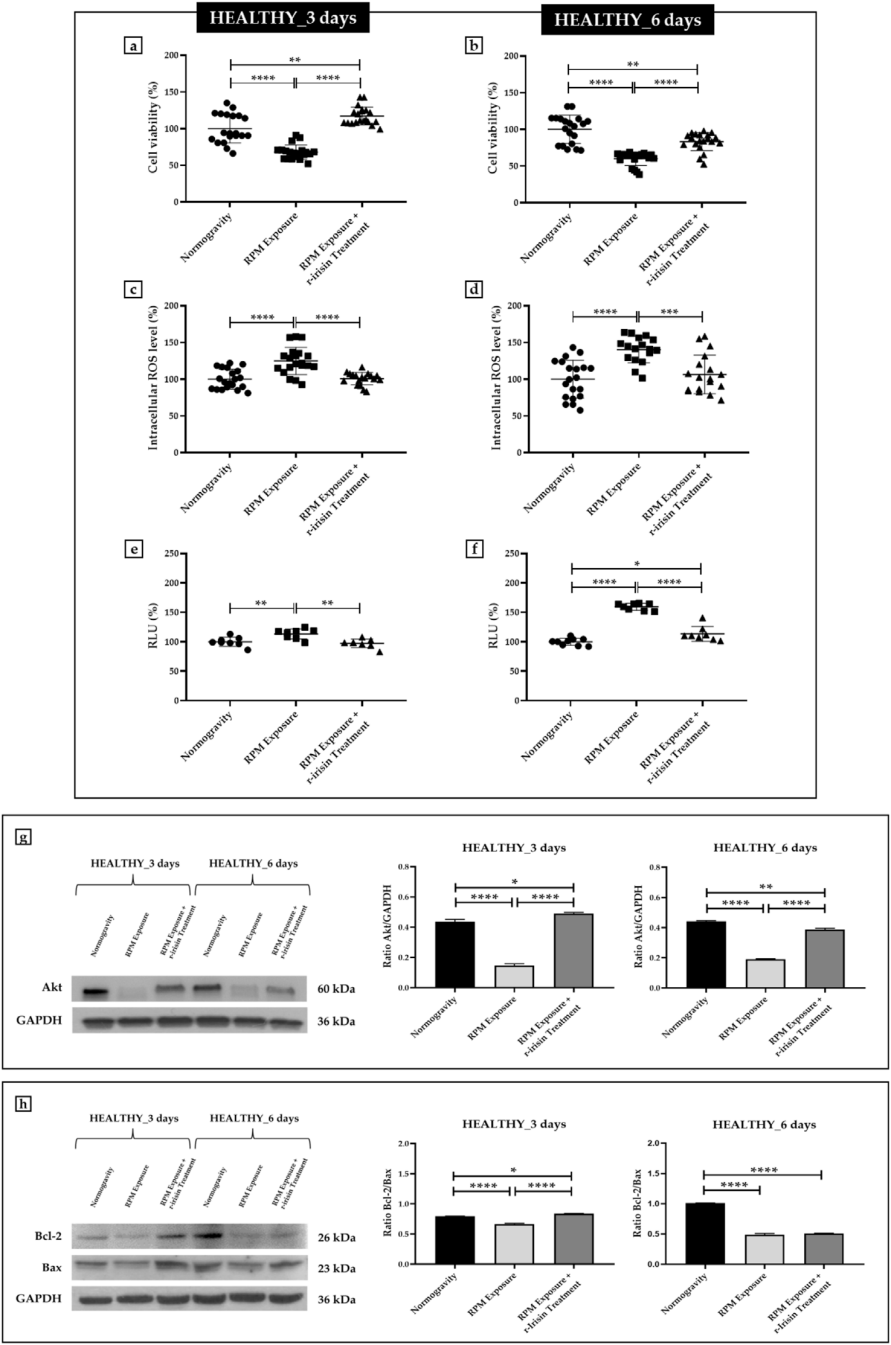
Effects of random positioning machine (RPM) exposure on the survival of primary osteoblast cultures derived from healthy (HEALTHY) subjects. **(a,b)** MTS assay: cell viability was completely **(a)** or partially **(b)** preserved in cells treated with r-irisin and exposed to RPM. **(c,d)** Intracellular levels of reactive oxygen species (ROS): the increase in oxidative stress induced by exposure to RPM was counteracted by r-irisin treatment. **(e,f)** Caspase-3/7 activity: total **(e)** or partial **(f)** prevention of caspase activity induced by RPM exposure in r-irisin-treated cells. **(g)** Western blotting analysis for Akt/protein kinase B (PKB): r-irisin treatment fully (3 days) or partially (6 days) preserved Akt expression. **(h)** Western blotting analysis for B-cell lymphoma 2 (Bcl-2) and Bax: significant increase in the Bcl-2/Bax ratio after r-irisin treatment (3 days); alteration of the Bcl-2/Bax ratio after 6 days of RPM exposure and r-irisin treatment, in favor of Bax.

Osteoblasts derived from HEALTHY subjects showed a significant reduction in cell viability after exposure to 3 days of RPM compared to cells maintained under normogravity conditions. Surprisingly, RPM exposure in the presence of r-irisin completely preserved cell survival, as demonstrated by the significant increase in viability of the treated cells compared to those maintained in normogravity and those exposed to RPM (Figure 1A). Similarly, the viability of cells exposed to 6 days of RPM was drastically reduced compared to cells maintained under normal conditions. In this case, r-irisin treatment partially preserved cell viability, with values significantly higher than those of untreated cells exposed to RPM, but significantly lower than those of cells in normogravity (Figure 1B).

Measurement of intracellular ROS levels showed a significant increase in oxidative stress in cells exposed to 3 days of RPM compared to those maintained under normal regimen. These values were even more pronounced after 6 days of RPM exposure compared to the control condition. Surprisingly, r-irisin treatment counteracted this increase in both experimental conditions, maintaining intracellular ROS levels at significantly lower values after both 3 days and 6 days of RPM exposure (Figures 1C, D).

In agreement, Figure 1E shows a significant increase in caspase-3/7 activity in osteoblasts exposed to 3 days of RPM compared to those maintained under normal conditions, which, however, is completely preserved by r-irisin treatment. Interestingly, the increase in RPM exposure time appeared to be closely related to the increase in caspase activity, as cells exposed to 6 days of RPM showed greater caspase activity compared to control cells. Surprisingly, the apoptosis increase was partially counteracted by r-irisin treatment, as the caspase-3/7 activity values were significantly lower than those of untreated cells exposed to RPM, but significantly higher than in the normogravity regime (Figure 1F).

These results were confirmed by Western blotting analysis with anti-Akt, anti-Bcl-2 and anti-Bax antibodies. In detail, Figure 1G shows a dramatic reduction in Akt expression in cells exposed to 3 days of RPM compared to those maintained under normal conditions. Surprisingly, r-irisin treatment completely preserved the expression of this protein, as kinase levels in treated cells were significantly higher than in cells in normogravity or exposed to RPM. As expected, Akt expression was also significantly reduced in cells exposed to 6 days of RPM compared to control cells. In this case, r-irisin treatment was not able to completely preserve Akt expression, as the values obtained from densitometric analysis were significantly higher than those of untreated cells exposed to RPM, but statistically lower than those of cells maintained under normogravity. In agreement, Figure 1H shows how different times of exposure to RPM modulate the ratio between anti-apoptotic and pro-apoptotic proteins differently. Indeed, the Bcl-2/Bax ratio is significantly reduced after exposure to 3 days of RPM compared to control cells. However, r-irisin treatment restored basal levels of Bcl-2 and Bax, with values significantly higher than in untreated cells exposed to RPM and to the normogravity condition. Not surprisingly, Bax expression was even more pronounced after 6-day RPM exposure, with values of the Bcl-2/Bax ratio significantly reduced compared to control cells. Unfortunately, no recovery was detected after r-irisin treatment, as the Bcl-2/Bax ratio values were statistically lower than those found under normogravity.

The mean values ± standard errors and their significance for the analyzed parameters are shown in Table 2.

**TABLE 2:** Mean values ± standard error and relative significance for the cell survival assessment in HEALTHY subjects after 3 and 6 days of RPM exposure.

		HEALTHY_3 days	Significance	HEALTHY_6 days	Significance
**Cell viability (%)**	Normogravity	100.0 ± 4.4	Normogravity vs. RPM Exposure, ****p < 0.0001; Normogravity vs. RPM Exposure + r-irisin Treatment, **p < 0.01; RPM Exposure vs. RPM Exposure + r-irisin Treatment, ****p < 0.0001; F = 58.9	100.0 ± 4.3	Normogravity vs. RPM Exposure, ****p < 0.0001; Normogravity vs. RPM Exposure + r-irisin Treatment, **p < 0.01; RPM Exposure vs. RPM Exposure + r-irisin Treatment, ****p < 0.0001; F = 40.1
	RPM Exposure	67.9 ± 2.2		59.9 ± 2.1	
	RPM Exposure + r-irisin Treatment	117.1 ± 2.7		83.3 ± 2.7	
**Intracellular ROS level (%)**	Normogravity	100.0 ± 3.0	Normogravity vs. RPM Exposure, ****p < 0.0001; RPM Exposure vs. RPM Exposure + r-irisin Treatment, ****p < 0.0001; F = 19.3	100.0 ± 5.8	Normogravity vs. RPM Exposure, ****p < 0.0001; RPM Exposure vs. RPM Exposure + r-irisin Treatment, ***p < 0.001; F = 14.6
	RPM Exposure	124.7 ± 4.1		140.2 ± 4.3	
	RPM Exposure + r-irisin Treatment	100.6 ± 2.0		106.4 ± 6.2	
**RLU (%)**	Normogravity	100.0 ± 2.8	Normogravity vs. RPM Exposure, **p < 0.01; RPM Exposure vs. RPM Exposure + r-irisin Treatment, **p < 0.01; F = 9.4	100.0 ± 2.0	Normogravity vs. RPM Exposure, ****p < 0.0001; Normogravity vs. RPM Exposure + r-irisin Treatment, *p < 0.05; RPM Exposure vs. RPM Exposure + r-irisin Treatment, ****p < 0.0001; F = 108.3
	RPM Exposure	113.2 ± 2.9		159.6 ± 2.1	
	RPM Exposure + r-irisin Treatment	97.2 ± 2.6		113.4 ± 4.5	
**Akt expression**	Normogravity	0.44 ± 0.02	Normogravity vs. RPM Exposure, ****p < 0.0001; Normogravity vs. RPM Exposure + r-irisin Treatment, *p < 0.05; RPM Exposure vs. RPM Exposure + r-irisin Treatment, ****p < 0.0001; F = 247.6	0.44 ± 0.01	Normogravity vs. RPM Exposure, ****p < 0.0001; Normogravity vs. RPM Exposure + r-irisin Treatment, **p < 0.01; RPM Exposure vs. RPM Exposure + r-irisin Treatment, ****p < 0.0001; F = 446.0
	RPM Exposure	0.15 ± 0.01		0.19 ± 0.01	
	RPM Exposure + r-irisin Treatment	0.49 ± 0.01		0.39 ± 0.01	
**Bcl-2/Bax ratio expression**	Normogravity	0.79 ± 0.01	Normogravity vs. RPM Exposure, ****p < 0.0001; Normogravity vs. RPM Exposure + r-irisin Treatment, *p < 0.05; RPM Exposure vs. RPM Exposure + r-irisin Treatment, ****p < 0.0001; F = 122.4	1.01 ± 0.01	Normogravity vs. RPM Exposure, ****p < 0.0001; Normogravity vs. RPM Exposure + r-irisin Treatment, ****p < 0.0001; F = 478.0
	RPM Exposure	0.66 ± 0.01		0.49 ± 0.02	
	RPM Exposure + r-irisin Treatment	0.84 ± 0.01		0.51 ± 0.01	

#### 3.2.2 OP patients

**FIGURE 2:**
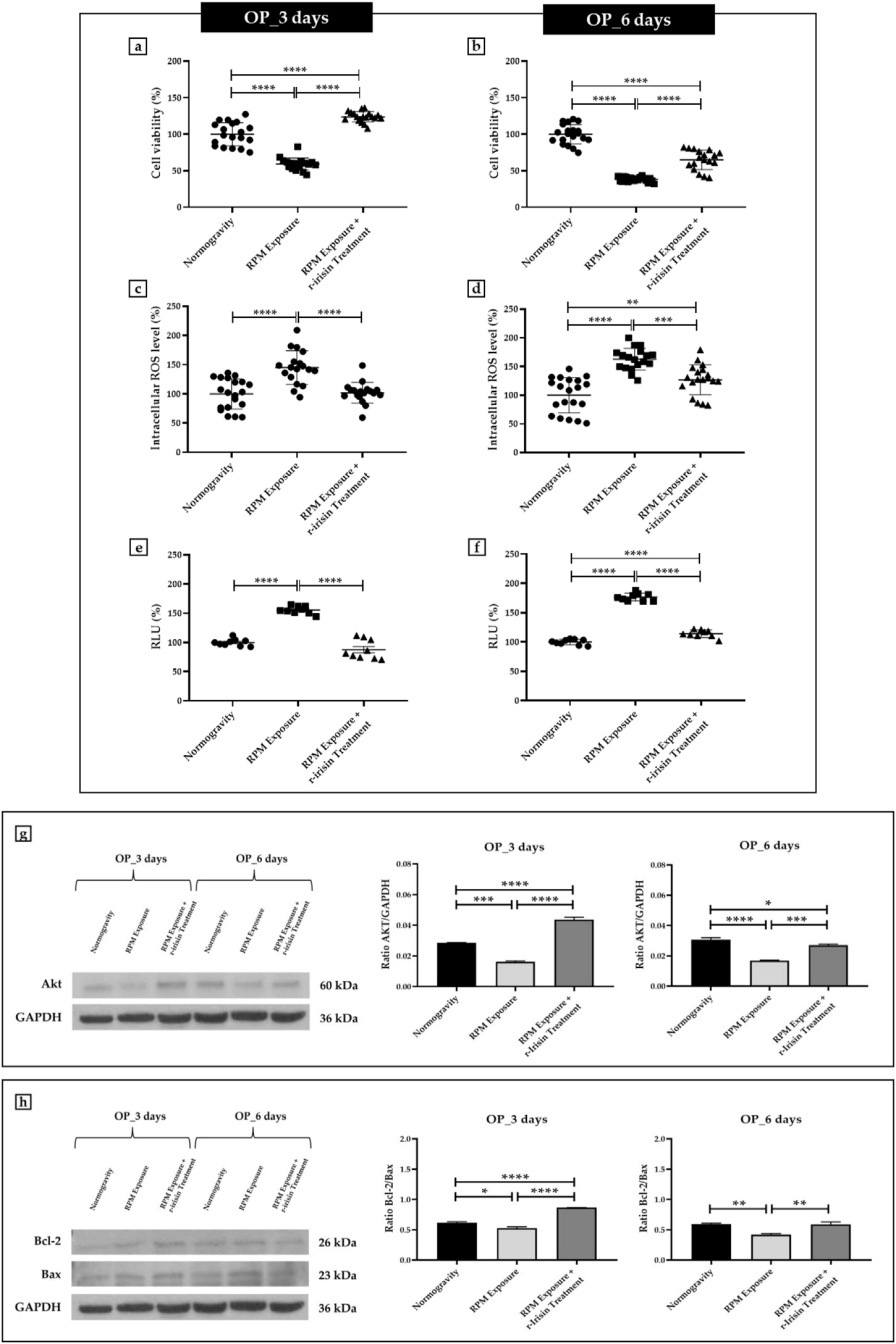
Effects of random positioning machine (RPM) exposure on the survival of primary osteoblast cultures derived from osteoporotic (OP) patients. **(a,b)** MTS assay: cell viability was completely **(a)** or partially **(b)** preserved in cells treated with r-irisin and exposed to RPM. **(c,d)** Intracellular levels of reactive oxygen species (ROS): the increase in oxidative stress induced by exposure to RPM was completely **(c)** or partially **(d)** counteracted by r-irisin treatment. **(e,f)** Caspase-3/7 activity: total **(e)** or partial **(f)** prevention of caspase activity induced by RPM exposure in r-irisin-treated cells. **(g)** Western blotting analysis for Akt/protein kinase B (PKB): r-irisin treatment completely (3 days) or partially (6 days) preserved Akt expression. **(h)** Western blotting analysis for B-cell lymphoma 2 (Bcl-2) and Bax: significant increase in Bcl-2/Bax ratio after r-irisin treatment (3 days); restoration of basal levels of Bcl-2 and Bax after 6 days of RPM exposure and r-irisin treatment.

Osteoblasts derived from OP patients showed a significant reduction in cell viability after exposure to 3 days of RPM compared to cells maintained under normal conditions. However, this effect was completely counteracted by r-irisin treatment, as cell viability values were significantly higher compared to cells maintained in normogravity and those exposed to RPM (Figure 2A). Exposure to 6 days of RPM further reduced cell viability, with statistically lower values compared to the normogravity regime. In this case, r-irisin treatment partially preserved cell viability, with values significantly higher than those of untreated cells exposed to RPM, but significantly lower than those of cells in normogravity (Figure 2B).

A significant increase in oxidative stress was observed by measuring intracellular ROS levels in cells exposed to 3 days of RPM compared to those maintained under normogravity. However, this effect was completely counteracted by r-irisin treatment, with significantly lower values compared to cells exposed to RPM (Figure 2C). Not surprisingly, oxidative stress was even more pronounced after 6 days of RPM exposure than in the control condition. In this case, r-irisin treatment partially counteracted this increase, maintaining intracellular ROS levels at significantly lower values compared to RPM-exposed cells, but significantly higher compared to the normogravity regime (Figure 2D).

In agreement, a significant increase in caspase-3/7 activity was found in osteoblasts exposed to 3 days of RPM compared to the normogravity condition, which was completely counteracted by r-irisin treatment (Figure 2E). The apoptosis increase was even more pronounced after 6 days of RPM exposure, with caspase-3/7 activity values significantly higher than in the control condition. Interestingly, r-irisin treatment partially counteracted this effect, as the caspase-3/7 activity values were significantly lower than those of untreated cells exposed to RPM, but significantly higher than in the normogravity regime (Figure 2F).

Figure 2G shows Western blotting analysis with anti-Akt antibodies. As expected, a significant reduction in Akt expression was detected in cells exposed to 3 days of RPM compared to those maintained under normogravity. Surprisingly, r-irisin treatment completely preserved the protein expression, with significantly higher values compared to control cells and those exposed to RPM. Akt expression was also drastically reduced in cells exposed to 6 days of RPM compared to the normogravity condition. In this case, r-irisin treatment partially preserved Akt expression, with kinase values significantly higher than those of untreated cells exposed to RPM, but statistically lower than those of cells maintained in normogravity. A Western blotting analysis for anti-Bcl-2 and anti-Bax antibodies is shown in Figure 2H, with significant differences depending on the experimental condition. In detail, a significant reduction in the Bcl-2/Bax ratio was observed compared to the normogravity regime, both after 3 days and 6 days of RPM exposure. Surprisingly, r-irisin treatment completely restored the ratio of anti-apoptotic to pro-apoptotic proteins after 3 days of RPM exposure, with Bcl-2/Bax ratio values significantly higher than both control and untreated cells exposed to RPM. Noteworthy, r-irisin treatment promoted recovery even after 6-day RPM exposure, with Bcl-2/Bax ratio values significantly higher than those observed in RPM-exposed cells.

The mean values ± standard errors and their significance for the analyzed parameters are shown in Table 3.

**TABLE 3:** Mean values ± standard error and relative significance for the cell survival assessment in OP patients after 3 and 6 days of RPM exposure.

		OP_3 days	Significance	OP_6 days	Significance
**Cell viability (%)**	Normogravity	100.0 ± 3.7	Normogravity vs. RPM Exposure, ****p < 0.0001; Normogravity vs. RPM Exposure + r-irisin Treatment, ****p < 0.0001; RPM Exposure vs. RPM Exposure + r-irisin Treatment, ****p < 0.0001; F = 156.5	100.0 ± 2.9	Normogravity vs. RPM Exposure, ****p < 0.0001; Normogravity vs. RPM Exposure + r-irisin Treatment, ****p < 0.0001; RPM Exposure vs. RPM Exposure + r-irisin Treatment, ****p < 0.0001; F = 153.4
	RPM Exposure	59.2 ± 1.9		38.2 ± 0.7	
	RPM Exposure + r-irisin Treatment	123.8 ± 1.67		64.9 ± 3.2	
**Intracellular ROS level (%)**	Normogravity	100.0 ± 5.8	Normogravity vs. RPM Exposure, ****p < 0.0001; RPM Exposure vs. RPM Exposure + r-irisin Treatment, ****p < 0.0001; F = 19.6	100.0 ± 6.9	Normogravity vs. RPM Exposure, ****p < 0.0001; Normogravity vs. RPM Exposure + r-irisin Treatment, **p < 0.01; RPM Exposure vs. RPM Exposure + r-irisin Treatment, ***p < 0.001; F = 27.9
	RPM Exposure	144.9 ± 6.8		162.9 ± 4.4	
	RPM Exposure + r-irisin Treatment	101.8 ± 4.2		126.9 ± 6.0	
**RLU (%)**	Normogravity	100.0 ± 2.0	Normogravity vs. RPM Exposure, ****p < 0.0001; RPM Exposure vs. RPM Exposure + r-irisin Treatment, ****p < 0.0001; F = 100.4	100.0 ± 1.5	Normogravity vs. RPM Exposure, ****p < 0.0001; Normogravity vs. RPM Exposure + r-irisin Treatment, ****p < 0.0001; RPM Exposure vs. RPM Exposure + r-irisin Treatment, ****p < 0.0001; F = 426.0
	RPM Exposure	155.7 ± 2.2		176.6 ± 2.2	
	RPM Exposure + r-irisin Treatment	87.6 ± 5.5		114.2 ± 2.1	
**Akt expression**	Normogravity	0.03 ± 0.00	Normogravity vs. RPM Exposure, ***p < 0.001; Normogravity vs. RPM Exposure + r-irisin Treatment, ****p < 0.0001; RPM Exposure vs. RPM Exposure + r-irisin Treatment, ****p < 0.0001; F = 195.1	0.03 ± 0.01	Normogravity vs. RPM Exposure, ****p < 0.0001; Normogravity vs. RPM Exposure + r-irisin Treatment, *p < 0.05; RPM Exposure vs. RPM Exposure + r-irisin Treatment, ***p < 0.001; F = 73.53
	RPM Exposure	0.02 ± 0.00		0.02 ± 0.00	
	RPM Exposure + r-irisin Treatment	0.04 ± 0.01		0.03 ± 0.00	
**Bcl-2/Bax ratio expression**	Normogravity	0.62 ± 0.02	Normogravity vs. RPM Exposure, *p < 0.05; Normogravity vs. RPM Exposure + r-irisin Treatment, ****p < 0.0001; RPM Exposure vs. RPM Exposure + r-irisin Treatment, ****p < 0.0001; F = 115.04	0.59 ± 0.01	Normogravity vs. RPM Exposure, **p < 0.01; RPM Exposure vs. RPM Exposure + r-irisin Treatment, **p < 0.01; F = 13.99
	RPM Exposure	0.53 ± 0.02		0.42 ± 0.02	
	RPM Exposure + r-irisin Treatment	0.87 ± 0.01		0.59 ± 0.03	

### 3.3 RPM exposure influences the mineralization process and PTX3 expression in primary human osteoblastic cells

The effects of two different RPM exposure times on the mineralization process in primary cultures of human osteoblasts from HEALTHY subjects and OP patients were evaluated by analysis of the PTX3 expression, a positive regulator of bone mineralization, by immunocytochemistry and Western blotting.

#### 3.3.1 HEALTHY subjects

**FIGURE 3:**
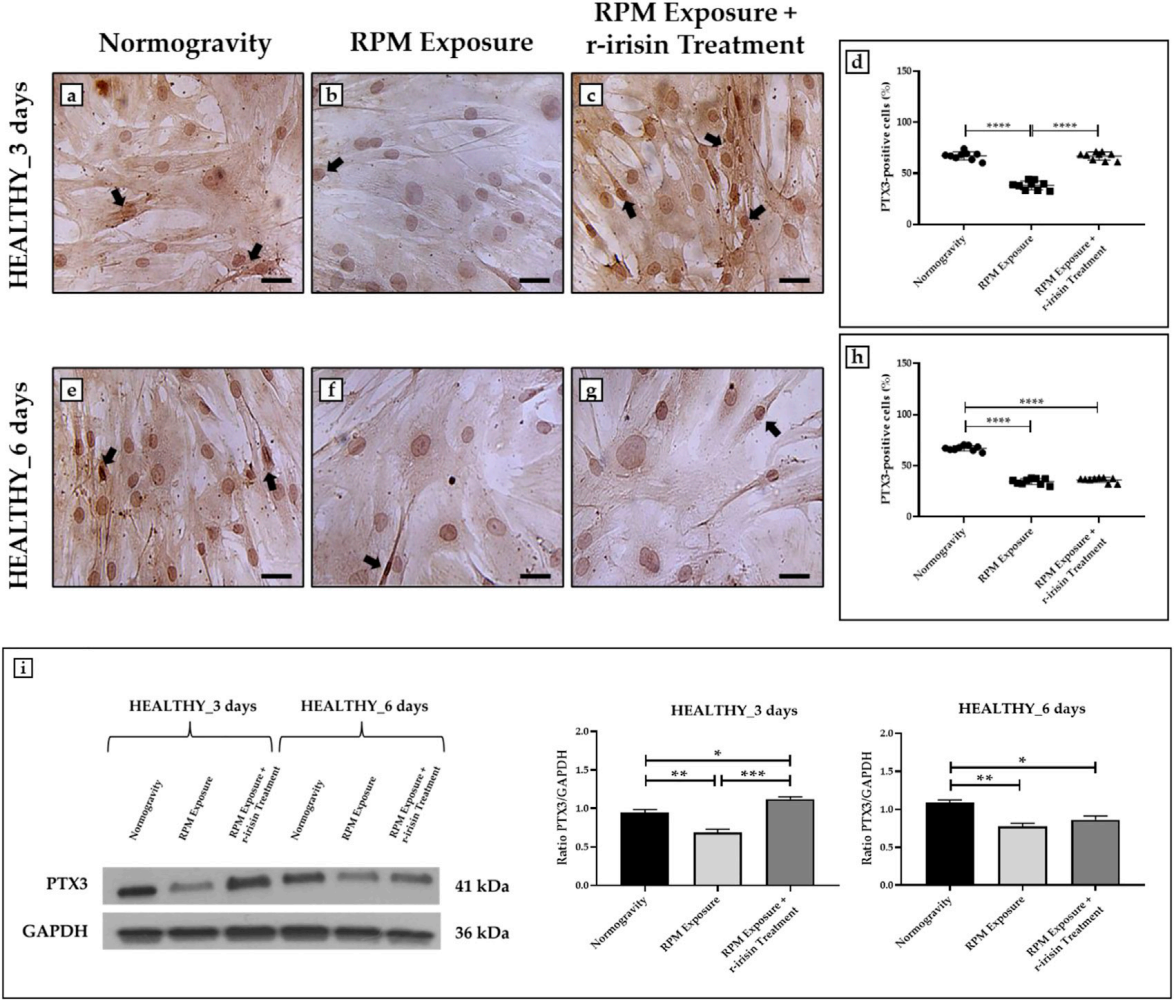
Effects of random positioning machine (RPM) exposure on the mineralization process in primary osteoblast cultures derived from healthy (HEALTHY) subjects. **(a–h)** Immunocytochemical analysis for PTX3: **(a–d)** significant increase in PTX3 expression (arrows) in cells exposed to 3 days of RPM and treated with r-irisin; **(e–h)** PTX3 expression (arrows) was not preserved by r-irisin treatment after 6 days of RPM exposure. **(i)** Western blotting analysis for PTX3: increased PTX3 expression in r-irisin-treated cells (3 days); PTX3 expression was not preserved by r-irisin treatment in cells exposed to 6 days of RPM. Images were magnified × 20, scale bar represents 50 μm.

The PTX3 expression was detected by immunocytochemical analysis under all experimental conditions, although with differences between groups, expressing PTX3-positive cells as a percentage of the total analyzed. Particularly, Figures 3A–D shows a significant reduction in PTX3 expression in cells exposed to 3-day RPM compared to those maintained in normogravity. Interestingly, r-irisin treatment prevents this effect, with significantly higher PTX3 expression values compared to cells exposed to RPM. Even in cells exposed to 6 days of RPM, a drastic reduction in PTX3 expression was found compared to the normogravity condition. Unfortunately, in this case, r-irisin treatment was not sufficient to restore PTX3 expression, as its levels were significantly lower than those of control (Figures 3E–H).

The immunocytochemistry data were confirmed by Western blotting analysis, which showed a positive band at around 41 kDa corresponding to the molecular weight of monomeric PTX3 in the protein extracts of all cell samples, although with differences between groups (Figure 3I). Indeed, a significant reduction in PTX3 expression was observed in cells exposed to RPM compared to control cells, both at 3 days and 6 days. Noteworthy, a very intense signal was detected in cells exposed to 3 days of RPM and treated with r-irisin, with significantly higher values compared to cells maintained in normogravity or exposed to RPM. Unfortunately, PTX3 expression was not preserved by r-irisin treatment when the exposure time to RPM was longer, with significantly lower values compared to control cells.

The mean values ± standard errors and their significance for the analyzed parameters are shown in Table 4.

**TABLE 4:** Mean values ± standard error and relative significance for the mineralization process assessment in HEALTHY subjects after 3 and 6 days of RPM exposure.

		HEALTHY_3 days	Significance	HEALTHY_6 days	Significance
**PTX3-positive cells (%)**	Normogravity	67.2 ± 1.3	Normogravity vs. RPM Exposure, ****p < 0.0001; RPM Exposure vs. RPM Exposure + r-irisin Treatment, ****p < 0.0001; F = 154.3	66.9 ± 0.8	Normogravity vs. RPM Exposure, ****p < 0.0001; Normogravity vs. RPM Exposure + r-irisin Treatment, ****p < 0.0001; F = 446.9
	RPM Exposure	38.1 ± 1.4		34.6 ± 0.9	
	RPM Exposure + r-irisin Treatment	66.9 ± 1.3		36.2 ± 0.8	
**PTX3 expression**	Normogravity	0.95 ± 0.04	Normogravity vs. RPM Exposure, **p < 0.01; Normogravity vs. RPM Exposure + r-irisin Treatment, *p < 0.05; RPM Exposure vs. RPM Exposure + r-irisin Treatment, ***p < 0.001; F = 33.1	1.09 ± 0.03	Normogravity vs. RPM Exposure, **p < 0.01; Normogravity vs. RPM Exposure + r-irisin Treatment, *p < 0.05; F = 14.73
	RPM Exposure	0.69 ± 0.04		0.77 ± 0.04	
	RPM Exposure + r-irisin Treatment	1.12 ± 0.03		0.86 ± 0.05	

#### 3.3.2 OP patients

**FIGURE 4:**
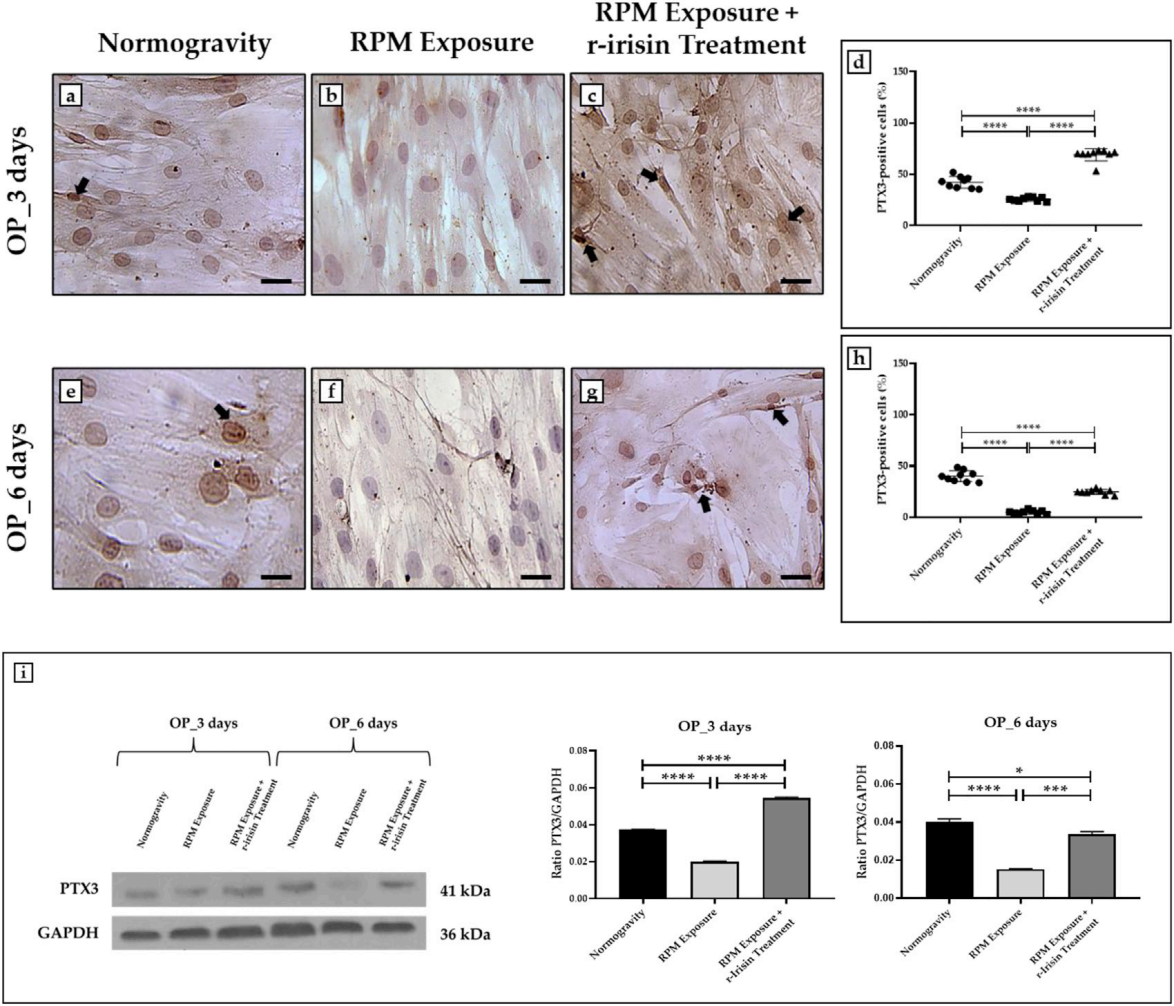
Effects of random positioning machine (RPM) exposure on the mineralization process in primary osteoblast cultures derived from osteoporotic (OP) patients. **(a–h)** Immunocytochemical analysis for PTX3: **(a–d)** significant increase in PTX3 expression (arrows) in cells exposed to 3 days of RPM and treated with r-irisin; **(e–h)** PTX3 expression (arrows) was not completely preserved by r-irisin treatment after 6 days of RPM exposure. **(i)** Western blotting analysis for PTX3: increased PTX3 expression in r-irisin-treated cells (3 days); PTX3 expression was not completely by r-irisin treatment in cells exposed to 6 days of RPM. Images were magnified × 20, scale bar represents 50 μm.

Immunocytochemical analysis revealed marked differences in PTX3 expression depending on the experimental group. Indeed, Figures 4A–D shows a significant reduction in PTX3 expression in cells exposed to 3 days of RPM compared to those maintained in normogravity. However, this effect was completely counteracted by r-irisin treatment, with significantly higher PTX3 expression values compared to RPM-exposed cells and control cells. After 6-day exposure to RPM, the PTX3 expression reduction was even more pronounced, with values almost undetectable compared to those found in normogravity. Not surprisingly, r-irisin treatment only partially preserved PTX3 expression, with values significantly lower than those of the control, but significantly higher than those of the cells exposed to RPM (Figures 4E–H).

In agreement, Western blotting analysis showed a positive band at around 41 kDa, corresponding to the molecular weight of monomeric PTX3, in the protein extracts of almost all cell samples (Figure 4I). Notably, a significant reduction in PTX3 expression was observed in cells exposed to 3 days of RPM compared to the normogravity condition. However, r-irisin treatment surprisingly preserved the mineralizing capacity of the cells, with significantly higher values than those observed in untreated cells exposed to RPM and in control cells. As expected, PTX3 expression was almost undetectable in cells exposed to 6 days of RPM, with significantly lower values compared to the normogravity regime. In this case, r-irisin treatment partially preserved PTX3 expression, with values significantly lower than in control cells, but statistically higher than in cells exposed to RPM.

The mean values ± standard errors and their significance for the analyzed parameters are shown in Table 5.

**TABLE 5:** Mean values ± standard error and relative significance for the mineralization process assessment in OP patients after 3 and 6 days of RPM exposure.

		OP_3 days	Significance	OP_6 days	Significance
**PTX3-positive cells (%)**	Normogravity	42.2 ± 1.9	Normogravity vs. RPM Exposure, ****p < 0.0001; Normogravity vs. RPM Exposure + r-irisin Treatment, ****p < 0.0001; RPM Exposure vs. RPM Exposure + r-irisin Treatment, ****p < 0.0001; F = 176.6	40.0 ± 1.8	Normogravity vs. RPM Exposure, ****p < 0.0001; Normogravity vs. RPM Exposure + r-irisin Treatment, ****p < 0.0001; RPM Exposure vs. RPM Exposure + r-irisin Treatment, ****p < 0.0001; F = 225.4
	RPM Exposure	25.7 ± 0.6		4.9 ± 0.6	
	RPM Exposure + r-irisin Treatment	63.8 ± 1.7		24.6 ± 0.8	
**PTX3 expression**	Normogravity	0.04 ± 0.00	Normogravity vs. RPM Exposure, ****p < 0.0001; Normogravity vs. RPM Exposure + r-irisin Treatment, ****p < 0.0001; RPM Exposure vs. RPM Exposure + r-irisin Treatment, ****p < 0.0001; F = 1736.0	0.04 ± 0.00	Normogravity vs. RPM Exposure, ****p < 0.0001; Normogravity vs. RPM Exposure + r-irisin Treatment, *p < 0.05; RPM Exposure vs. RPM Exposure + r-irisin Treatment, ***p < 0.001; F = 109.7
	RPM Exposure	0.02 ± 0.00		0.02 ± 0.00	
	RPM Exposure + r-irisin Treatment	0.05 ± 0.00		0.03 ± 0.01	

## 4 Discussion

Our results show how RPM exposure produces a marked effect on bone tissue cells. The reproduction of the biological effects of microgravity, as well as the exacerbation of the osteoporotic condition, produces an increase in cell death and a reduction in the mineralizing capacity of osteoblasts isolated from participants undergoing hip arthroplasty for high-energy fracture or fragility, respectively. Although a similar RPM exposure response trend was observed in both groups, it is important to note that all measurements performed on HEALTHY cells were characterized by considerably higher baseline values than those obtained from OP cells. This difference is attributable to the age of the participants and the health status of the bone tissue, highlighting the need to conduct parallel analyses to investigate the mechanisms underlying bone loss under different conditions. Interestingly, a single administration of r-irisin has been shown to be a useful tool to counteract cell death and mineralization deficits induced by RPM exposure, suggesting its potential use in the development of strategies to counteract bone mass loss induced by weightlessness or osteoporosis. However, the action of r-irisin in counteracting the cellular damage induced by RPM exposure appears to persist for a limited period, probably due to the substance’s short half-life (Tsiani et al., 2021), highlighting the need for further studies to identify an optimal treatment strategy based on the use of r-irisin and/or investigate the efficacy of alternative strategies to be used in a complementary manner to r-irisin.

### 4.1. Effects of RPM exposure on human primary osteoblast cell death and r-irisin efficacy

Osteoblasts from the HEALTHY group were used to reproduce the bone mass loss that occurs in astronauts exposed to spaceflight. Our results clearly showed that RPM exposure leads to significant alterations in cell survival, intracellular ROS levels, caspase activity and expression of survival and cell death proteins. In agreement, these alterations were more pronounced after RPM exposure for 6 days, confirming that the extent of microgravity-induced damage is proportional to exposure time (Pani et al., 2013).

One of our objectives was to test whether a single administration of r-irisin could counteract these effects, so that it could be suggested as a preventive strategy for the bone mass loss that affects astronauts during spaceflight. Surprisingly, a single administration of r-irisin was sufficient to prevent apoptotic cell death, as assessed by caspase 3/7 activity, Akt expression and the Bcl-2/Bax ratio, demonstrating both the efficacy of r-irisin and the reversibility of damage induced by RPM exposure. However, these effects were not observed after RPM exposure for 6 days, as r-irisin did not completely restore the stress-induced alterations. Indeed, although an improvement in terms of cell viability, caspase activity and cell survival/death protein expression were observed, r-irisin did not provide complete protection against the damage induced by RPM exposure, suggesting the need for its continuous administration.

Interestingly, the bone mass loss is a condition that also characterizes the elderly with osteoporosis and, in this context, the use of r-irisin has already been suggested as a potential therapeutic tool (Baran et al., 2022). However, the mechanism by which this hormone preserves the bone mass loss due to osteoporosis has not been well elucidated, nor have the molecular pathways common to the weightlessness-induced loss of bone mass and the pathological condition been determined. In this regard, osteoblasts from OP patients were exposed to RPM to exacerbate the osteoporotic condition and identify molecular mediators common to both conditions. In general, basal cell survival levels, as well as Akt expression and Bcl-2/Bax ratio, were lower than those observed in cells from the HEALTHY group, while intracellular ROS and caspase activity values were significantly increased. RPM exposure for 3 days induced a significant alteration of the examined cell survival/death parameters, which were dramatically impaired following a 6-day exposure. Thus, the efficacy of a single administration of r-irisin was investigated to test whether this substance could prevent the apoptotic death that results in bone mass loss in osteoporosis (Badr Roomi et al., 2021; Zerlotin et al., 2022). Again, r-irisin was able to prevent the alterations induced by exposure to 3 days of RPM, confirming both the involvement of an apoptotic pathway in the osteoporosis progression and the hormone’s extraordinary efficacy in counteracting the disease (Xu et al., 2020). However, in the case of 6-day exposure, a single administration of r-irisin was not sufficient to prevent RPM-induced damage, suggesting the need for repeated administrations or the use of additional tools to complement r-irisin.

Overall, our results highlight the efficacy of r-irisin in preventing cell death of primary human osteoblasts induced by RPM exposure. This evidence agrees with the observations of Storlino et al., who demonstrated the efficacy of r-irisin in preventing apoptosis in the MLO-Y4 cell line (Storlino et al., 2020). Particularly, cleavage of caspase-3 induced by 1 μM dexamethasone was prevented by a 24-h treatment with r-irisin. Furthermore, a sustained increase in the Bcl-2/Bax ratio in the cortical bone of osteoporotic r-irisin-treated mice was found, confirming the extraordinary anti-apoptotic power of this myokine (Storlino et al., 2020). In agreement, Zhao and others reported that irisin can influence osteoblastic cell proliferation through the regulation of the Akt/β-catenin signalling pathway, which is known to be a crucial modulator of the effects of mechanical deformation (Zhao et al., 2021).

However, our results indicate that the r-irisin efficacy depends on the time of exposure to the insult, as cell viability is not fully preserved for periods longer than 3 days. Noteworthy, r-irisin treatment also improved, at least partially, cell survival characteristics in the OP group, suggesting its potential role in counteracting osteoporosis progression. Therefore, further studies are needed to better understand the influence of r-irisin on Akt regulation the cell death process.

### 4.2 Effects of RPM exposure on human primary osteoblast mineralization and r-irisin efficacy

Bone loss, known to afflict both astronauts exposed to spaceflight and osteoporotic patients, is characterized by a significant reduction in the mineralizing capacity of bone cells and loss of expression of important bone formation markers (Cristofaro et al., 2019; Coulombe et al., 2020). In this context, one of the main objectives of this study was to evaluate changes in the PTX3 expression pattern of osteoblasts exposed to RPM and to assess whether a single administration of r-irisin could influence this phenomenon. As expected, cells in the HEALTHY group exposed to RPM for 3 and 6 days showed altered PTX3 expression. However, a single administration of r-irisin counteracted the mineralization deficit in cells exposed to RPM for 3 days, whereas it was ineffective after a 6-day treatment. These observations suggest that a single administration of r-irisin produces protective effects that are maintained for a limited period, not guaranteeing complete protection when exposed to RPM for longer periods.

Mineralization defects are a typical condition of osteoporosis, and evidence concerning altered expression patterns of bone markers in this disease is plentiful (Ruden et al., 2018; Liu et al., 2020). Indeed, r-irisin, due to its unique ability to regulate bone metabolism, has already been proposed as a potential strategy to prevent and/or counteract the onset of osteoporosis (Zerlotin et al., 2022). However, there is a need to further investigate the beneficial effects of this hormone on the health of the bone tissue of patients with osteoporosis, who may suffer prolonged bed rest and severe worsening of the disease state due to adverse events. In this regard, osteoblasts from the OP group exposed to RPM for 3 days and 6 days showed a significant PTX3 expression loss. A single administration of r-irisin significantly improved PTX3 expression in cells exposed to 3 days of RPM, but the effects of this administration were not observed for a 6-day exposure period. Therefore, r-irisin has the potential to be a solution to the conditions leading to bone loss and mineralization defects.

In this regard, Colaianni et al. (2017) demonstrated the r-irisin efficacy in preserving and restoring bone mass in mice with suspended hind limbs, suggesting its possible use for both preventive and curative purposes. In agreement, Zhu and others observed lower bone density in FNDC5/irisin-deficient mice, concomitant with delayed bone development and mineralization compared to control animals (Zhu et al., 2021). Furthermore, low-dose r-irisin administration in young mice has been reported to increase cortical BMD, confirming the role of irisin as a positive regulator of bone formation and mineralization (Cariati et al., 2021b). Overall, the beneficial effects of irisin on bone tissue are well known and widely documented. However, further studies are needed to understand the full potential of this surprising protein to develop optimal strategies to counteract the bone loss that occurs in astronauts exposed to spaceflight, as well as in sedentary, elderly and/or osteoporotic subjects.

## 5 Conclusion

The use of r-irisin could represent a promising strategy to prevent apoptotic death of osteoblasts and subsequent demineralization of bone tissue, as demonstrated by the total protection observed after exposure to 3 days of RPM. However, the persistence of its effects on cellular wellbeing appears to be limited over time, as a single administration was not sufficient to completely prevent the damage induced by a 6-day RPM exposure. We do not exclude the possibility that repeated administrations of r-irisin may offer complete protection from cell death even after RPM exposures of more than 3 days. Alternatively, r-irisin administration could be supplemented with additional defence tools to achieve longer-lasting protective effects. Therefore, the development of an effective formula based on the use of this protein could not only preserve the bone mass of astronauts exposed to microgravity, but also counteract the progression of osteoporosis and improve the bone quality of sedentary subjects.

## 6 Limits of the study

The limitation of this study is the use of RPM as a model to simulate the biological effects of microgravity, as the constant agitation of the culture chambers may favour the flow shear phenomenon and influence the experimental results. To minimize this phenomenon, all experiments were conducted in the complete absence of air bubbles in the culture chamber. Furthermore, it should be made clear that RPM does not simulate microgravity, but reproduces the biological effects induced by it. Therefore, all experiments conducted using RPM should be reproduced under real microgravity conditions.

## Data Availability

The original contributions presented in the study are included in the article/Supplementary Material, further inquiries can be directed to the corresponding author.
