# Supplementary material for: Recombinant irisin prevents cell death and mineralization defects induced by random positioning machine exposure in primary cultures of human osteoblasts: A promising strategy for the osteoporosis treatment
Source: Front Physiol. 2023 Mar 15;14:1107933. doi: 10.3389/fphys.2023.1107933 (PMC10052411; doi:10.3389/fphys.2023.1107933)
Supplement: Supplementary file 1 [file DataSheet1.docx]

Supplementary Material


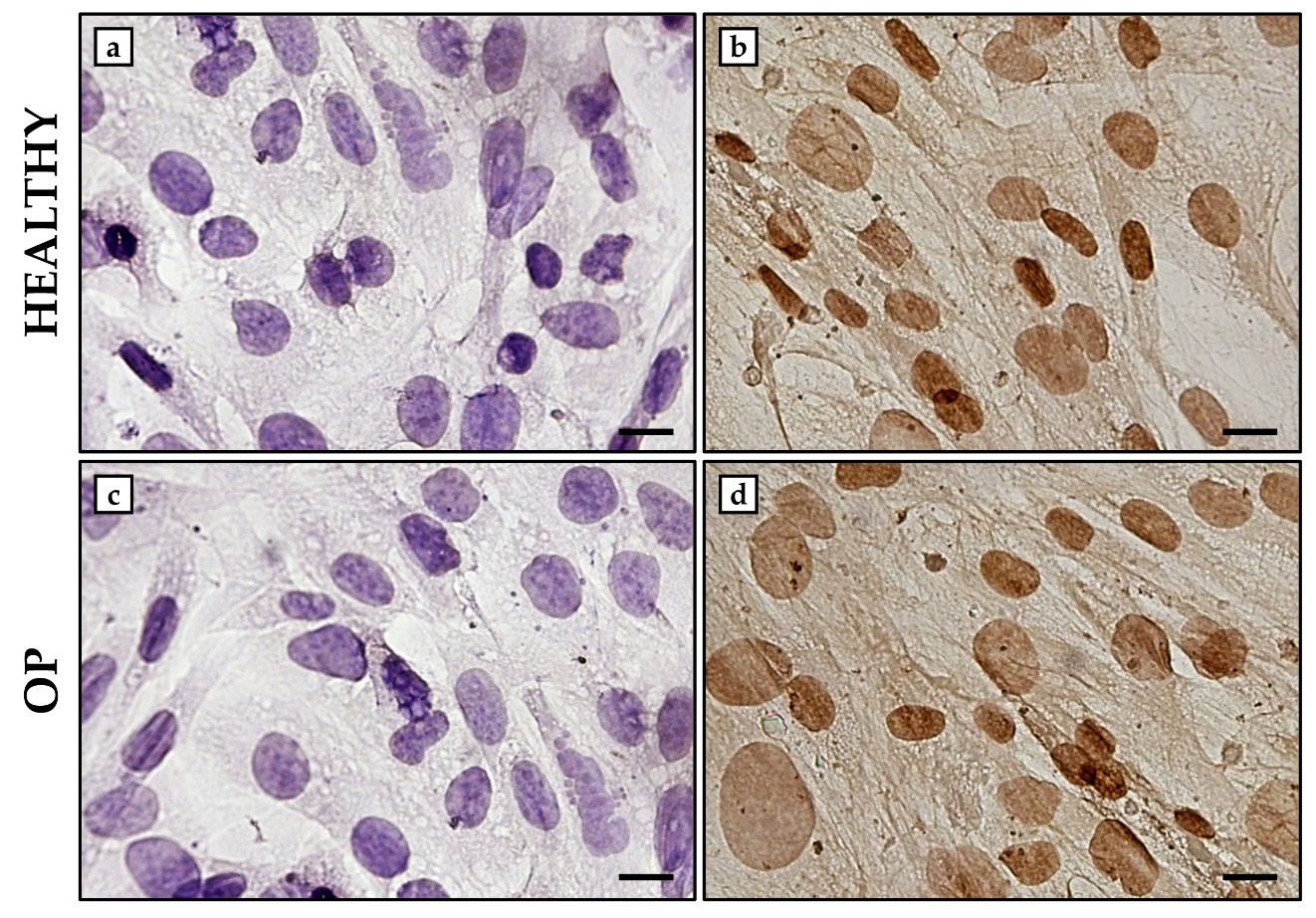


**Figure S1. Characterization of primary cultures of human osteoblasts.** The runt-related transcription factor 2 (RUNX2) expression by immunocytochemistry was evaluated to characterize primary cultures of osteoblasts from healthy subjects (HEALTHY) and osteoporotic patients (OP). (**a**) HEALTHY osteoblasts used as negative control. (**b**) HEALTHY osteoblasts incubated with anti-RUNX2 antibody. (**c**) OP osteoblasts used as a negative control. (**d**) OP osteoblasts incubated with anti-RUNX2 antibody. Images were magnified 40×, scale bar represents 20 μm.


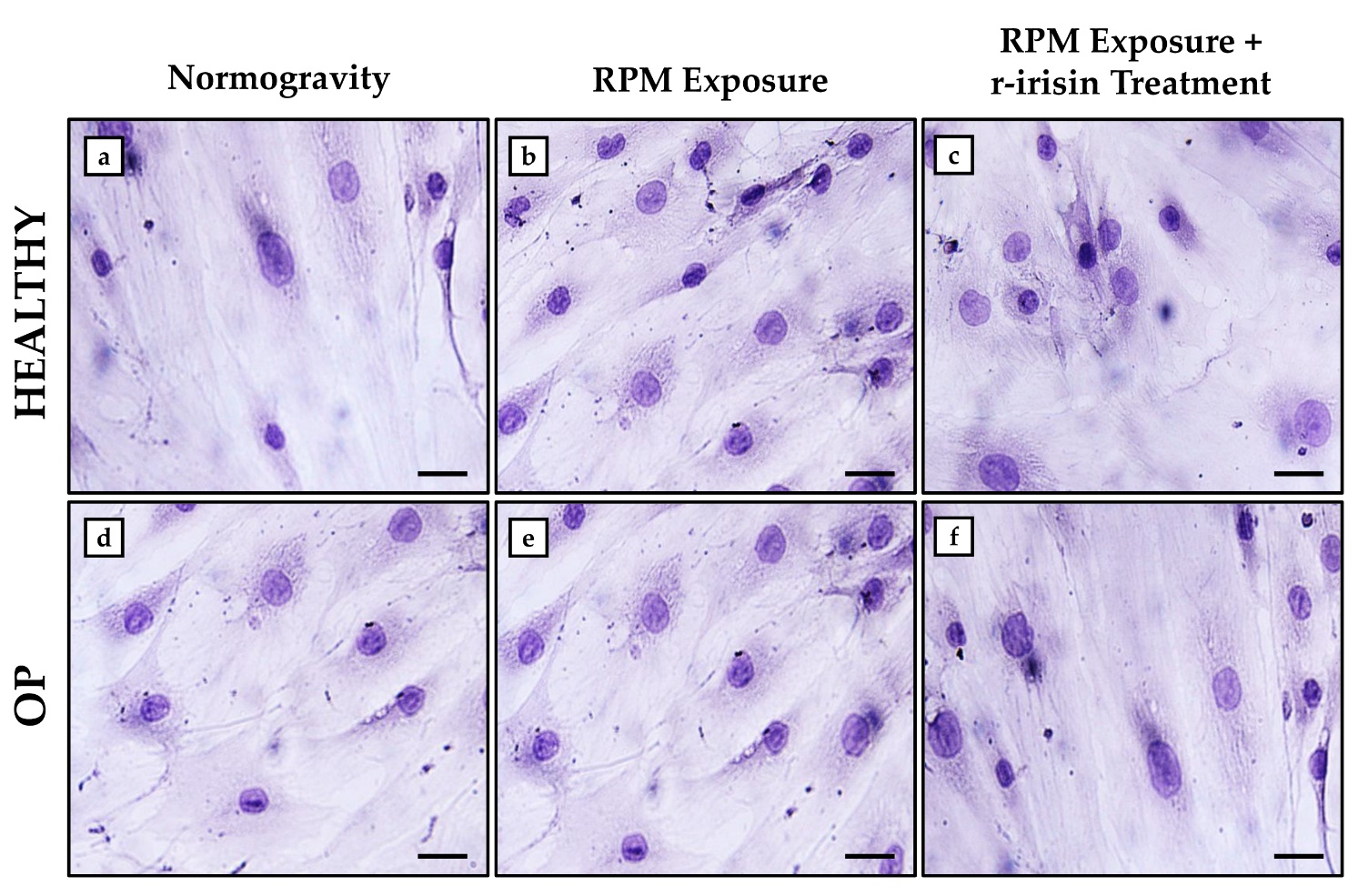


**Figure S2.** **Immunocytochemistry analysis for Pentraxin 3 (PTX3) expression in primary cultures of human osteoblasts.** (**a**) PTX3-negative control in HEALTHY subjects undergoing normogravity regimen. (**b**) PTX3-negative control in HEALTHY subjects undergoing RPM exposure. (**c**) PTX3-negative control in HEALTHY subjects undergoing RPM exposure in association to recombinant irisin (r-irisin) treatment. (**d**) PTX3-negative control in OP patients undergoing normogravity regimen. (**e**) PTX3-negative control in OP patients undergoing RPM exposure. (**f**) PTX3-negative control in OP patients undergoing RPM exposure in association to r-irisin treatment. Images were magnified 40×, scale bar represents 20 μm.


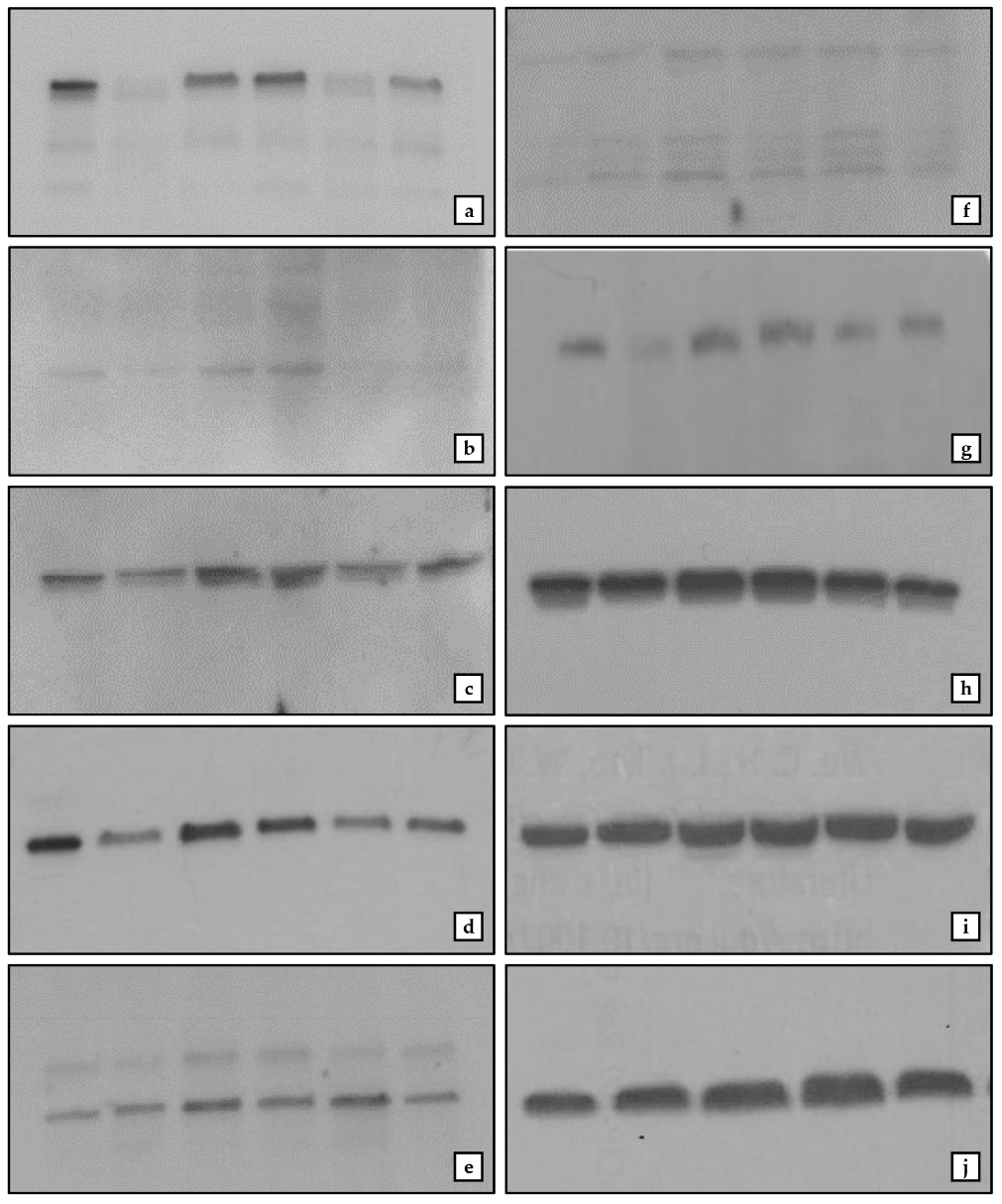


**Figure S3. Original western blotting images.** (**a**) Akt expression (molecular weight 60 kDa) in HEALTHY subjects. (**b**) Bcl-2 expression (molecular weight 26 kDa) in HEALTHY subjects. (**c**) Bax expression (molecular weight 23 kDa) in HEALTHY subjects. (**d**) PTX3 expression (molecular weight 41 kDa) in HEALTHY subjects. (**e**) Akt expression (molecular weight 60 kDa) in OP patients. (**f**) Bcl-2 (molecular weight 26 kDa) and Bax (molecular weight 23 kDa) expression in OP patients. (**g**) PTX3 expression (molecular weight 41 kDa) in OP patients. (**h**–**j**) GAPDH expression (molecular weight 36 kDa) in HEALTHY subjects and OP patients.
